# Regulation-Focused Psychotherapy for Children (RFP-C): Advances in the Treatment of ADHD and ODD in Childhood and Adolescence

**DOI:** 10.3389/fpsyg.2020.572917

**Published:** 2020-11-03

**Authors:** Mariagrazia Di Giuseppe, Tracy A. Prout, Timothy Rice, Leon Hoffman

**Affiliations:** ^1^Department of Surgical, Medical and Molecular Pathology, Critical and Care Medicine, University of Pisa, Pisa, Italy; ^2^Ferkauf Graduate School of Psychology, Yeshiva University, New York, NY, United States; ^3^Icahn School of Medicine at Mount Sinai, Columbia University, New York, NY, United States; ^4^Pacella Research Center of New York Psychoanalytic Society and Institute, New York, NY, United States

**Keywords:** emotion regulation, defense mechanisms, psychotherapy, oppositional defiant disorder, RFP-C, DMRS, externalizing behavior

## Introduction

Externalizing behaviors are among the most common problems of childhood and affect many aspects of psychological development (Wilens et al., 2002; Liu, 2004). Children with oppositional defiant disorder (ODD) are at higher risk for developing emotional disorders as well as conduct disorder and antisocial personality disorder in adulthood, especially when they receive inadequate psychological support (Rutter et al., 2006; Stringaris and Goodman, 2009; Diamantopoulou et al., 2010; Reef et al., 2010; Hudson et al., 2018).

Parental stress, depression, and anxiety are elevated among parents of children with ODD as compared to those with typical developmental patterns (Katzmann et al., 2018; Lin et al., 2019; Manti et al., 2019). For many years, behavioral parent training (BPT) approaches, including Parent Management Training (PMT), have been the primary treatment option for children with ODD because of their robust evidence base for children with externalizing behaviors (Serketich and Dumas, 1996; Brestan and Eyberg, 1998; Kazdin and Weisz, 1998). All BPT interventions rely on traditional cognitive behavioral strategies in working primarily with the parent, and include behavioral modeling, rewards, reinforcement, and developmentally-appropriate consequences for misbehavior (Webster-Stratton, 1994; Eyberg and Bussing, 2010). A limitation associated with behavioral parent programs is elevated attrition rates for vulnerable populations affected by factors, such as low socioeconomic status, ethnic minority status, low parental functioning, high maternal stress, low parental motivation, and high child symptom severity (Kazdin, 1990; Werba et al., 2006; Fernandez and Eyberg, 2009; Lanier et al., 2011; Granero et al., 2015). Attrition in behavioral parent training may also be due to common parental attributions about where the problem resides—within the child (Baden and Howe, 1992; Bickett et al., 1996; Prout et al., 2015). Parents may feel that since the treatment approach is through the parents, they are implicitly responsible for the child's maladaptive behavior, and may avoid sustained engagement in the treatment to unburden themselves of heightened feelings of responsibility or blame. Finally, behavioral approaches usually do not directly identify, address, or engage with the underlying emotions in the child, which can become dysregulated. An inability to effectively address and engage with these emotions can lead to persistent oppositional behaviors.

In recent years, L.H., T.R., and T.A.P. developed a novel, manualized, time-limited psychodynamic treatment approach for children who present with disruptive behaviors and emotional dysregulation named Regulation-Focused Psychotherapy for Children (RFP-C; Hoffman and Rice, 2016). RFP-C conceptualizes children's externalizing behaviors as expressions of maladaptive defense mechanisms formulated as the products of developmental delays in the implicit emotion regulation system (Rice and Hoffman, 2014). RFP-C targets the strengthening of the child's implicit emotional regulation system through direct work on the child's maladaptive defenses and provides psychoeducation and empathic support to parents of the child in distress. Throughout 16 individual play therapy sessions and four parent meetings, the clinician increases understanding that all behavior, especially disruptive behavior, has meaning in the service of emotional and behavioral regulation (Prout et al., 2019a). This insight leads to a decreased need and reliance to act on the distressing emotions (e.g., less need for disruptive behaviors) and an increased ability to tolerate, work through, and talk about the feelings that previously needed to be warded off. In addition, parents are relieved of the burden of feeling heightened responsibility as the locus of the child's problems. The clinician joins the parent and the child as a system all directly working toward improvement. The efficacy of RFP-C has been demonstrated in an initial pilot study (Prout et al., 2019b) and promising preliminary data from a recent randomized controlled trial of the intervention (Di Giuseppe et al., 2020c; Prout, 2020).

## Assessment of Defense Mechanisms in Children

Defined as unconscious operations that protect the self from the awareness of feelings and thoughts of internal conflicts and external stressors (Vaillant, 1992; MacGregor and Olson, 2005; American Psychiatric Association, 2013), defense mechanisms play a key role in RFP-C. This therapeutic approach is based on the observation, interpretation, and developing awareness of child defense mechanisms either activated “in session” or reported in the patient narratives (Perry et al., 2020). The accuracy of defense mechanism assessment becomes essential for successfully addressing immature defensive patterns and fostering adaptive implicit emotion regulation (Di Giuseppe et al., 2019, 2020a). Despite progress in defense mechanism assessment in adults (Bond et al., 1989; Perry, 1990; Perry and Henry, 2004; Di Giuseppe et al., 2014, 2020b), only a few measures assess defenses in children (Cramer, 1991; Laor et al., 2001; Nimroody et al., 2019). None of these utilize an empirically-derived, observer-rated methodology that can be applied to psychotherapy sessions.

To fill the lack of empirical measures for child defense mechanisms assessment, one of the authors (M.D.G.), developed the Q-sort version of the Perry's Defense Mechanisms Rating Scale (DMRS-Q; Di Giuseppe et al., 2014) for clinical use. Our aim is to create a new computerized observer-rated measure for assessing defense mechanisms in children, the Defense Mechanisms Rating Scale—Q-Sort for Children (DMRS-QC), based on the theoretical background of the DMRS-Q. This will be the first attempt to provide an empirical instrument consistent with the definitions and hierarchical organization of defense mechanisms (Vaillant, 1992; American Psychiatric Association, 1994; Perry and Henry, 2004). Analyzing defense mechanisms in action in RFP-C has the potential to promote identification of the defensive profile of children with disruptive behaviors, as well as the changes that underlie successful RFP-C treatment outcome. The DMRS-QC will provide an effective and easy-to-use measure for examining defense mechanisms in children across a wide range of treatment modalities.

## Training in RFP-C

One of the advantages of RFP-C is the ease with which it can be applied. As any other evidence-based psychotherapy, RFP-C requires a specific training for its reliable use.

The training includes didactic instruction, a competency quiz, and attendance at several supervision sessions. RFP-C therapists learn how to focus on behavioral, non-verbal, verbal, and play disruptions as evidence of defense mechanisms in action. Attention is also paid to the importance of the therapeutic relationship as a vehicle for therapeutic intervention. The recognition of specific defensive patterns and their underlying regulation function during the session allow the RFP-C therapist to efficiently address the implicit emotion regulation strategies and enhance changes in the child overall defensive maturity. Thus, the knowledge of definitions and functions of defense mechanisms is a crucial part of the RFP-C training.

## Discussion

Preliminary validation studies on the efficacy of RFP-C in treating ODD children have found that the treatment provides relief from symptoms of ODD and an increase in overall emotion regulation (Prout et al., 2019b; Prout, 2020). In working with parents, therapists help them in observing, reflecting and understanding the triggers which provoke the child's disruptive behavior. Parents can then reflect of more effective ways of addressing the triggers. Working with children who have ODD allows them to find new ways of thinking about their emotions and behaviors as a defensive response to anger, frustration, and fear. Throughout the therapeutic relationship children experience positive social relationships where unpleasant feelings can be thought about and not only acted upon.

Initially formulated in New York City as a collaboration among faculty from three institutions, The New York Psychoanalytic Society and Institute, The Icahn School of Medicine at Mount Sinai, and most importantly at the Ferkauf Graduate School of Psychology where the randomized controlled trial has been conducted, RFP-C is now practiced by many practitioners, who have had various exposures to the principles of RFP-C across the United States. Close collaboration with one of the authors (M.D.G.) has allowed us to expand the dissemination of this manualized psychotherapy to Italy, where the Center for Regulation Focused Psychotherapy for Children will begin to offer official RFP-C training in the near future.

## Author Contributions

MD conceived the idea and made a significant contribution by drafting the manuscript. All authors critically revised the manuscript and approved the final version to be published.

## Conflict of Interest

The authors declare that the research was conducted in the absence of any commercial or financial relationships that could be construed as a potential conflict of interest.

## References

[B1] American Psychiatric Association (1994). Diagnostic and Statistical Manual of Mental Disorders. 4th Edn. Washington, DC: American Psychiatric Association.

[B2] American Psychiatric Association (2013). Diagnostic and Statistical Manual of Mental Disorders. 5th Edn. Washington, DC: American Psychiatric Association.

[B3] Baden A. D. Howe G. W. (1992). Mothers' attributions and expectancies regarding their conduct disordered children. J. Abnorm. Child Psychol. 20, 467–485. 10.1007/BF00916810 1487591

[B4] Bickett L. R. Milich R. Brown R. T. (1996). Attributional styles of aggressive boys and their mothers. J. Abnorm. Child Psychol. 24, 457–472. 10.1007/BF01441568 8886942

[B5] Bond M. Perry J. C. Gautier M. Goldenberg M. Oppenheimer J. Simand J. (1989). Validating the self-report of defense styles. J. Pers. Disord. 3, 101–112.

[B6] Brestan E. V. Eyberg S. M. (1998). Effective psychosocial treatments of conduct-disordered children and adolescents: 29 years, 82 studies, and 5,272 kids. J. Clin. Child Psychol. 27, 180–189. 10.1207/s15374424jccp2702_5 9648035

[B7] Cramer P. (Ed.). (1991). “The defense mechanism manual,” in The Development of Defense Mechanisms (New York, NY: Springer-Verlag), 215–234.

[B8] Di Giuseppe M. Gennaro A. Lingiardi V. Perry J. C. (2019). The role of defense mechanisms in emerging personality disorders in clinical adolescents. Psychiatry 82, 128–142. 10.1080/00332747.2019.1579595 30925112

[B9] Di Giuseppe M. Perry J. C. Conversano C. Gelo O. C. G. Gennaro A. (2020a). Defense mechanisms, gender and adaptiveness in emerging personality disorders in adolescent outpatients. J. Nerv. Ment. Dis. 10.1097/NMD.0000000000001230. [Epub ahead of print]. 32947450

[B10] Di Giuseppe M. Perry J. C. Lucchesi M. Michelini M. Vitiello S. Piantanida A. (2020b). Preliminary validity and reliability of the novel self-report based on the Defense Mechanisms Rating Scales (DMRS-SR-30). Front. Psychiatry 11:870 10.3389/fpsyt.2020.00870 33005160 PMC7479239

[B11] Di Giuseppe M. Perry J. C. Petraglia J. Janzen J. Lingiardi V. (2014). Development of a Q-Sort version of the Defense Mechanism Rating Scales (DMRS-Q) for clinical use. J. Clin. Psychol. 70, 452–465. 10.1002/jclp.22089 24706519

[B12] Di Giuseppe M. Prout T. A. Fabiani M. Kui T. (2020c). Defensive profile of parents of children with externalizing problems receiving regulation-focused psychotherapy for children (RFP-C): a pilot study. Mediterr. J. Clin. Psychol. 8 10.6092/2282-1619/mjcp-2515

[B13] Diamantopoulou S. Verhulst F. C. van der Ende J. (2010). Testing developmental pathways to antisocial personality problems. J. Abnorm. Child Psychol. 38, 91–103. 10.1007/s10802-009-9348-7 19688258 PMC2809948

[B14] Eyberg S. M. Bussing R. (2010). “Parent-child interaction therapy for preschool children with conduct problems,” in Clinical Handbook of Assessing and Treating Conduct Problems in Youth, eds Murrihy R. C. Kidman A. D. Ollendick T. H. (New York, NY: Springer), 139–162. 10.1007/978-1-4419-6297-3_6

[B15] Fernandez M. A. Eyberg S. M. (2009). Predicting treatment and follow-up attrition in parent-child interaction therapy. J. Abnorm. Child Psychol. 37, 431–441. 10.1007/s10802-008-9281-1 19096926

[B16] Granero R. Louwaars L. Ezpeleta L. (2015). Socioeconomic status and oppositional defiant disorder in preschoolers: parenting practices and executive functioning as mediating variables. Front. Psychol. 6:1412. 10.3389/fpsyg.2015.01412 26441784 PMC4585310

[B17] Hoffman L. Rice T. (2016). Manual of Regulation-Focused Psychotherapy for Children (RFP-C) With Externalizing Behaviors: A Psychodynamic Approach. New York, NY: Routledge.

[B18] Hudson J. L. Murayama K. Meteyard L. Morris T. Dodd H. F. (2018). Early childhood predictors of anxiety in early adolescence. J. Abnorm. Child Psychol. 47, 1121–1133. 10.1007/s10802-018-0495-6 30569254

[B19] Katzmann J. Döpfner M. Görtz-Dorten A. (2018). Child-based treatment of oppositional defiant disorder: mediating effects on parental depression, anxiety and stress. Eur. Child Adolesc. Psychiatry 27, 1181–1192. 10.1007/s00787-018-1181-5 29948233

[B20] Kazdin A. E. (1990). Premature termination from treatment among children referred for antisocial behavior. Child Psychol. Psychiatry Allied Discipl. 31, 415–425. 10.1111/j.1469-7610.1990.tb01578.x 2318922

[B21] Kazdin A. E. Weisz J. R. (1998). Identifying and developing empirically supported child and adolescent treatments. J. Consult. Clin. Psychol. 66, 19–36. 9489260 10.1037//0022-006x.66.1.19

[B22] Lanier P. Kohl P. L. Benz J. Swinger D. Moussette P. Drake B. (2011). Parent–child interaction therapy in a community setting: examining outcomes, attrition, and treatment setting. Res. Soc. Work Pract. 21, 689–698. 10.1177/1049731511406551 24839378 PMC4021486

[B23] Laor N. Wolmer L. Cicchetti D. V. (2001). The comprehensive assessment of defensive style: measuring defense mechanisms in children and adolescents. J. Nerv. Ment. Disord. 189, 360–368. 10.1097/00005053-200106000-00003 11434636

[B24] Lin X. Li Y. Xu S. Ding W. Zhou Q. Du H.. (2019). Family risk factors associated with oppositional defiant disorder symptoms, depressive symptoms, and aggressive behaviors among chinese children with oppositional defiant disorder. Front. Psychol. 10:2062. 10.3389/fpsyg.2019.02062 31611830 PMC6769082

[B25] Liu J. (2004). Childhood externalizing behavior: theory and implications. J. Child Adolesc. Psychiatr. Nurs. 17, 93–103. 10.1111/j.1744-6171.2004.tb00003.x 15535385 PMC1617081

[B26] MacGregor M. W. Olson T. R. (2005). “Defense mechanisms: their relation to personality and health. an exploration of defense mechanisms assessed by the Defense-Q,” in Advances in Psychology Research, Vol. 36, ed Columbus A. (Hauppauge, NY: Nova Science Publishers), 95–141.

[B27] Manti F. Giovannone F. Sogos C. (2019). Parental stress of preschool children with generalized anxiety or oppositional defiant disorder. Front. Pediatr. 7:415 10.3389/fped.2019.00415 31681713 PMC6811652

[B28] Nimroody T. Hoffman L. Christian C. Rice T. Murphy S. (2019). Development of a defense mechanisms manual for children's Doll Play (DMCP). J. Infant Child Adolesc. Psychother. 18, 58–70. 10.1080/15289168.2018.1565005

[B29] Perry J. C. (1990). Defense Mechanism Rating Scales (DMRS). 5th Edn. Cambridge, MA: J. C. Perry.

[B30] Perry J. C. Banon E. Bond M. (2020). Change in defense mechanisms and depression in a pilot study of antidepressive medications plus 20 sessions of psychotherapy for recurrent major depression. J. Nerv. Ment. Dis. 208, 261–268. 10.1097/NMD.0000000000001112 32221178

[B31] Perry J. C. Henry M. (2004). “Studying defense mechanisms in psychotherapy using the Defense Mechanism Rating Scales,” in Defense Mechanisms: Theoretical, Research and Clinical Perspectives, eds Hentschel U. Smith G. Draguns J. G. Ehlers W. (Amsterdam: Elsevier), 165–192.

[B32] Prout T. A. (2020). “Psychodynamic treatment for children and families: outcomes of a randomized controlled trial of RFP-C,” in Winter Meeting (New York, NY: American Psychoanalytic Association).

[B33] Prout T. A. Gerber L. E. Gaines E. Hoffman L. Rice T. R. (2015). The development of an evidence-based treatment: regulation-focused psychotherapy for children with externalizing disorders. J. Child Psychother. 41, 255–271. 10.1080/0075417X.2015.1090695

[B34] Prout T. A. Malone A. Rice T. Hoffman L. (2019a). Resilience, defenses, and implicit emotion regulation in psychodynamic child psychotherapy. J. Contemp. Psychother. 49, 235–244. 10.1007/s10879-019-09423-w

[B35] Prout T. A. Rice T. R. Murphy S. Gaines E. Aizin S. Sessler D.. (2019b). Why is it easier to get mad than it is to feel sad? Pilot study of regulation focused psychotherapy for children. Am. J. Psychother. 72, 2–8. 10.1176/appi.psychotherapy.20180027 30786738

[B36] Reef J. Diamantopoulou S. van Meurs I. Verhulst F. van der Ende J. (2010). Predicting adult emotional and behavioral problems from externalizing problem trajectories in a 24-year longitudinal study. Eur. Child Adolesc. Psychiatry 19, 577–585. 10.1007/s00787-010-0088-6 20140633

[B37] Rice T. R. Hoffman L. (2014). Defense mechanisms and implicit emotion regulation: a comparison of a psychodynamic construct with one from contemporary neuroscience. J. Am. Psychoanal. Assoc. 62, 693–708. 10.1177/0003065114546746 25082875

[B38] Rutter M. Kim-Cohen J. Maughan B. (2006). Continuities and discontinuities in psychopathology between childhood and adult life. J. Child Psychol. Psychiatry 47, 276–295. 10.1111/j.1469-7610.2006.01614.x 16492260

[B39] Serketich W. J. Dumas J. E. (1996). The effectiveness of behavioural parent training to modify antisocial behaviour in children: a meta-analysis. Behav. Ther. 27, 171–186. 10.1016/S0005-7894(96)80013-X

[B40] Stringaris A. Goodman R. (2009). Longitudinal outcome of youth oppositionality: irritable, headstrong, and hurtful behaviors have distinctive predictions. J. Am. Acad. Child Adolesc. Psychiatry 48, 404–412. 10.1097/CHI.0b013e3181984f30 19318881

[B41] Vaillant G. E. (1992). Ego Mechanisms of Defense: A Guide for Clinicians and Researchers. Washington, DC: American Psychiatric Press.

[B42] Webster-Stratton C. H. (1994). “Parent intervention content: typical questions,” in Troubled Families—Problem Children, eds Webster C. Stratton C. Herbert M. (Chicester: John Wiley and Sons), 237–308.

[B43] Werba B. E. Eyberg S. M. Boggs S. R. Algina J. (2006). Predicting outcome in parent-child interaction therapy: success and attrition. Behav. Modif. 30, 618–646. 10.1177/0145445504272977 16894233

[B44] Wilens T. E. Biederman J. Brown S. Tanguay S. Monuteaux M. C. Blake C.. (2002). Psychiatric comorbidity and functioning in clinically referred preschool children and school-age youths with ADHD. J. Am. Acad. Child Adolesc. Psychiatry 41, 262–268. 10.1097/00004583-200203000-00005 11886020

